# Converting Instance Checking to Subsumption: A Rethink for Object Queries over Practical Ontologies

**DOI:** 10.4236/ijis.2015.51005

**Published:** 2015-01

**Authors:** Jia Xu, Patrick Shironoshita, Ubbo Visser, Nigel John, Mansur Kabuka

**Affiliations:** 1 Department of Electrical and Computer Engineering, University of Miami, Coral Gables, USA; 2 Department of Computer Science, University of Miami, Coral Gables, USA

**Keywords:** Description Logic, Ontology, Object Query, SHI, Most Specific Concept

## Abstract

Efficiently querying Description Logic (DL) ontologies is becoming a vital task in various data-intensive DL applications. Considered as a basic service for answering object queries over DL ontologies, instance checking can be realized by using the most specific concept (MSC) method, which converts instance checking into subsumption problems. This method, however, loses its simplicity and efficiency when applied to large and complex ontologies, as it tends to generate very large MSCs that could lead to intractable reasoning. In this paper, we propose a revision to this MSC method for DL SHI, allowing it to generate much simpler and smaller concepts that are specific enough to answer a given query. With independence between computed MSCs, scalability for query answering can also be achieved by distributing and parallelizing the computations. An empirical evaluation shows the efficacy of our revised MSC method and the significant efficiency achieved when using it for answering object queries.

## 1. Introduction

Description logics (DLs) play an ever growing role in providing a formal and semantic-rich way to model and represent structured data in various applications, including semantic web, healthcare and biomedical research, etc. [1]. A knowledge base in description logic, usually referred to as a DL ontology, consists of an *assertional* component (ABox T) for data description, where *individuals* (single objects) are introduced and their mutual relationships are described using assertional axioms. Semantic meaning of the ABox data can then be unambiguously specified by a *terminological* component (TBox T) of the DL ontology, where abstract *concepts* and *roles* (binary relations) of the application domain are properly defined.

In various applications of description logics, one of the core tasks for DL systems is to provide an efficient way to manage and query the assertional knowledge (*i.e.* ABox data) in a DL ontology, especially for those data-intensive applications; and DL systems are expected to scale well with respect to (w.r.t.) the fast growing ABox data, in settings such as semantic webs or biomedical systems. The most basic reasoning service provided by existing DL systems for retrieving objects from ontology ABoxes is *instance checking*, which tests whether an individual is a member of a given concept. Instance retrieval (*i.e.* retrieve all instances of a given concept) then can be realized by performing a set of instance checking calls.

In recent years, considerable efforts have been dedicated to the optimization of algorithms for ontology reasoning and query answering [2]-[4]. However, due to the enormous amount of ABox data in realistic applications, existing DL systems, such as HermiT [4] [5], Pellet [6], Racer [7] and FaCT++ [8], still have difficulties in handling the large ABoxes, as they are all based on the (*hyper*) *tableau* algorithm that is computationally expensive for expressive DLs (e.g. up to EXPTIME for instance checking in DL SHIQ), where the complexity is usually measured in the size of the TBox, the ABox and the query [9]-[13]. In practice, since the TBox and the query are usually much smaller compared with the ABox, the reasoning efficiency could be mostly affected by the size of the ABox.

One of the solutions to this reasoning scalability problem is to develop a much more efficient algorithm that can easily handle large amount of ABox data. While another one is to reduce size of the data by either partitioning the ABox into small and independent fragments that can be easily handled in parallel by existing systems [14]-[16], or converting the ABox reasoning into a TBox reasoning task (*i.e.* ontology reasoning without an ABox), which could be “*somewhat*” independent of the data size, the TBox is static and relatively simple, as demonstrated in this paper.

A common intuition about converting instance checking into a TBox reasoning task is the so-called most specific concept (MSC) method [10] [17] [18] that computes the MSC of a given individual and reduces any instance checking of this individual into a *subsumption test* (*i.e.* test if one concept is more general than the other). More precisely, for a given individual, its most specific concept should summarize all information of the individual in a given ontology ABox, and should be specific enough to be subsumed by any concept that the individual belongs to. Therefore, once the most specific concept *C* of an individual *a* is known, in order to check if *a* is an instance of any given concept *D*, it is sufficient to test if *C* is subsumed by *D*. With the MSC of every individual in the ABox, the efficiency of online object queries can then be boosted by performing an offline classification of all MSCs that can pre-compute many instance checks [10]. Moreover, if a large ontology ABox consists of data with great diversity and isolation, using the MSC method for instance checking could be more efficient than the original ABox reasoning, since the MSC would have the tableau algorithm to explore only the related information of the given individual, potentially restricted to a small subset of the ABox. Also, this method allows the reasoning to be parallelized and distributed, since MSCs are independent of each other and each preserves complete information of the corresponding individual.

Despite these appealing properties possessed by the MSC method, the computation of a MSC could be difficult even for a very simple description logic such as ALE . The difficulty arises mainly from the support of qualified existential restrictions (e.g. ∃*R.C*) in DLs, such that when converting a role assertion (e.g. *R*(*a,b*)) of some individuals into an existential restriction, so that information of that given individual may not be preserved completely. For a simple example, consider converting assertions

R(a,b)andA(a)

into a concept for individual *a* . In this case, we can always find a more specific concept for *a* in the form of

$$A⊓\underset{n}{\underset{︸}{\exists R.\exists R.\cdots \exists R}}.A$$

by increasing *n* , and none of them would capture the complete information of individual *a* . Such information loss is due to the occurrence of cycles in the role assertions, and none of the existential restrictions in DL could impose a circular interpretation (model) unless *nominals* (e.g. {*a*}) are involved or (local) reflexivity is presented [5].

Most importantly, due to the support of existential restrictions, computation of the MSC for a given individual may involve assertions of other entities that are connected to it through role assertions. This implies not only the complexity of the computation for MSCs but also the potential that the resulting MSCs may have larger than desired sizes. In fact, in many of the practical ontology ABoxes (e.g. a social network or semantic webs), most of the individuals could be connected to each other through role assertions, forming a huge connected component in the ABox graph. Under this situation, the resulting MSC could be extremely large and reasoning with it may completely degenerate into an expensive reasoning procedure.

**Figure 1:**
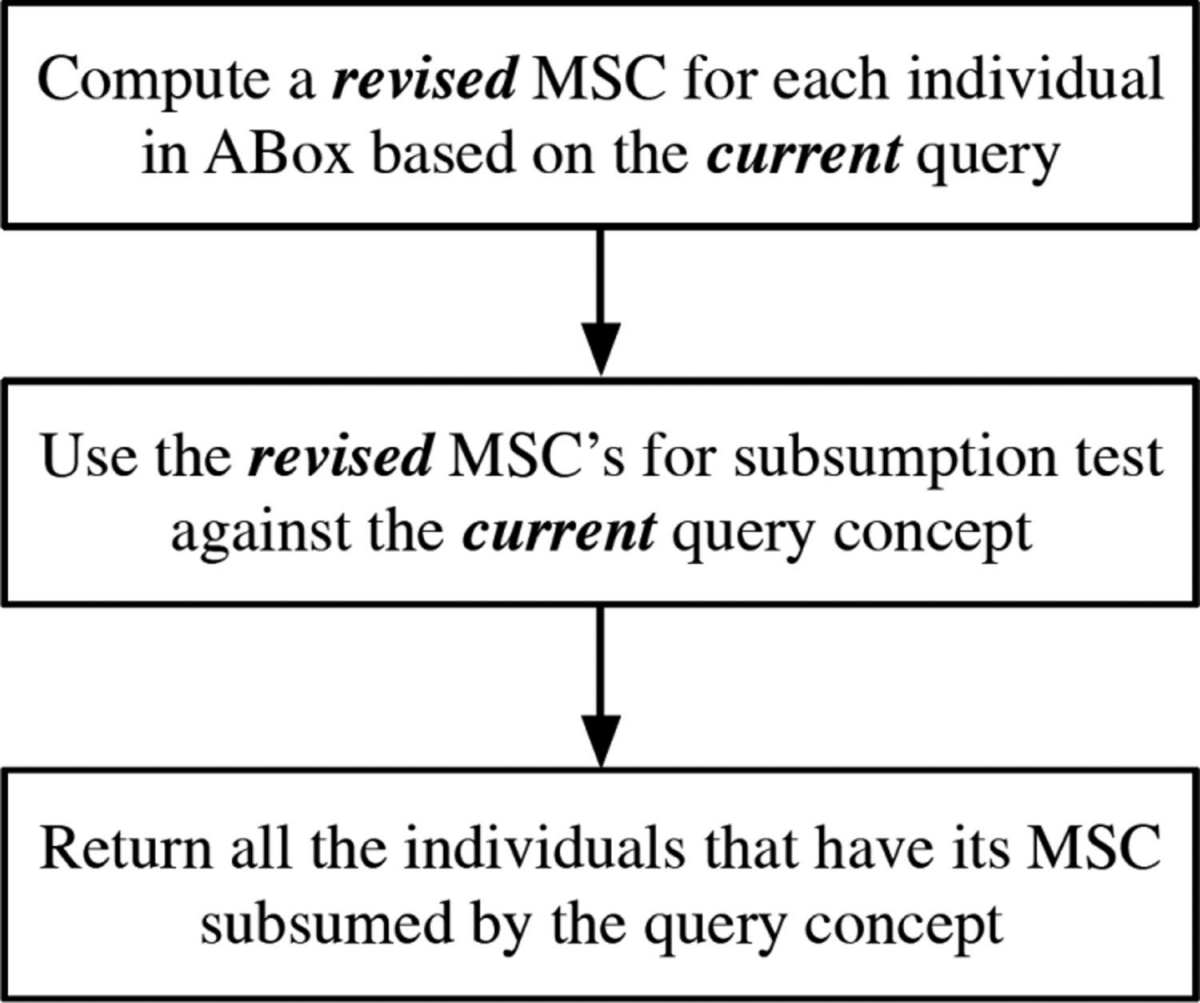
A procedure for instance retrieval for a given query based on our revised MSC method.

In this paper, we propose a revised MSC method for DL SHI that attempts to tackle the above mentioned problems, by applying a *call-by-need* strategy together with optimizations. That is, instead of computing the most specific concepts that could be used to answer any queries in the future, the revised method takes into consideration only the related ABox information with *current* query and computes a concept for each individual that is only *specific enough* to answer it w.r.t. the TBox. Based on this strategy, the revision allows the method to generate much simpler and smaller concepts than the original MSCs by ignoring irrelevant ABox assertions. On the other hand, the complexity reduction comes with the price of re-computation (*i.e.* online computation of MSCs) for every new coming query, if no optimization is applied. Nevertheless, as shown in our experimental evaluation, the simplicity achieved could be significant in many practical ontologies, and the overhead is thus negligible compared with the reasoning efficiency gained for each instance checking and query answering. Moreover, due to the re-computations, *we do not assume a static ontology or query*, and the ABox data is amenable to frequent modifications, such as insertion or deletion, which is in contrast to the original MSC method where a relatively static ABox is assumed. A procedure for instance retrieval based on our revised MSC method is shown in **Figure 1**.

The revised MSC method could be very useful for efficient instance checking in many practical ontologies, where the TBox is usually small and manageable while the ABox is in large scale as a database and tends to change frequently. Particularly, this method would be appealing to large ontologies in *non-Horn* DLs, where current optimization techniques such as rule-based reasoning or pre-computation may fall short. Moreover, the capability to parallelize the computation is another compelling reason to use this technique, in cases where answering object queries may demand thousands or even millions of instance checking tasks.

Our contributions in this paper are summarized as follows:

1) We propose a call-by-need strategy for the original MSC method, instead of computing the most specific concepts offline to handle any given query, which allows us to focus on the current queries and to generate online much smaller concepts that are sufficient to compute the answers. This strategy makes our MSC method suitable for query answering in ontologies, where frequent modifications to the ontology data are not uncommon;
2) We propose optimizations that can be used to further reduce sizes of computed concepts in practical ontologies for more efficient instance checking;
3) Finally, we evaluate our approach on a range of test ontologies with large ABoxes, including ones generated by existing benchmark tools and realistic ones used in biomedical research. The evaluation shows the efficacy of our proposed approach that can generate significantly smaller concepts than the original MSC. It also shows the great reasoning efficiency that can be achieved when using the revised MSC method for instance checking and query answering.

The rest of the paper is organized as follows: in Section 2, we introduce the background knowledge of a description logic and DL ontology; in Section 3, we give more detailed discussion about the MSC method and our call-by-need strategy; Section 4 presents the technical details of the revised MSC method; Section 5 discusses the related work; Section 6 presents an empirical evaluation on our proposed method; and finally, Section 7 concludes our work.

## 2. Preliminaries

The technique proposed in this paper is for description logic SHI . For technical reasons, we need a *constrained* use of nominals on certain conditions (*i.e.* assertion cycles), which requires logic SHIQ . Thus, in this section, we give a brief introduction to formal syntax and semantics of logic SHIO , DL ontologies, and basic reasoning tasks for derivation of logical entailments from DL ontologies.

### 2.1. Description Logic SHIO

The vocabulary of description logic SHIO includes a set **R** of named roles with a subset of **R**_+_ ⊆ **R** of transitive roles, a set **C** of named (atomic) concepts, and a set **I** of named individuals.

#### Definition 2.1 (SHIO Role)

*A role in* SHIO *is either a named (atomic) one R* ∈ **R** *or an inverse one R^-^ with R* ∈ **R**, *and the complete role set of* SHIO *can be denoted* R* = **R** ∪ {*R*^-^|*R* ∈ **R**}. To avoid role representation such as R^--^, a function Inv(·) *is defined, such that* Inv(*R*) = *R*^-^ *R*^-^ *if R is a role name, and* Inv(*R*) = *P if R* = *P*^-^ for some role name P . A role R is transitive, denoted Trans(*R*), *if either R or* Inv(*R*) belongs to R_+_ .

#### Definition 2.2 (SHIO Concept)

*A* SHIO-*concept is either an atomic* (*named*) concept or a complex one that can be defined using the following constructs recursively

C,D∷=A∣{o}∣T∣⊥∣¬C∣C⊓D∣C⊔D∣∀R.C∣∃R.C

where *A* is an atomic concept **C**, *o* in is a named individual, and *R* ∈ R*.

Description logic SHI is then defined as a fragment of SHIO , which disallows the use of nominal (*i.e.* {*o*}) as a construct for building complex concepts.

#### Definition 2.3 (SHIO Semantics)

*The meaning of an entity in* SHIO is defined by a model-theoretical semantics using an interpretation denoted $I=\left(\Delta^{I},.^{I}\right)$, *where* $\Delta^{I}$ *is referred to as a non-empty domain and* $.^{I}$ *is an interpretation function. The function* $.^{I}$ *maps every atomic concept in* **C** *to a subset of* $\Delta^{I}$, *every ABox individual to an element of* $\Delta^{I}$, *and every role to a subset of* $\Delta^{I}\times \Delta^{I}$. Interpretation for other concepts and role are given below:

$$\begin{array}{cc} & T^{I}=\Delta^{I}\\ & {⊥}^{I}=\emptyset \\ & {\left\{o\right\}}^{I}=\left\{o^{I}\right\}\\ & \neg C^{I}=\Delta^{I}\backslash C^{I}\\ & {\left(R^{-}\right)}^{I}=\left\{\left(y,x\right)|\left(x,y\right)\in R^{I}\right\}\\ & {\left(C⊓D\right)}^{I}=C^{I}\cap D^{I}\\ & {\left(C⊔D\right)}^{I}=C^{I}\cup D^{I}\\ & {\left(\exists R.C\right)}^{I}=\left\{x|\exists y.\left(x,y\right)\in R^{I}\wedge y\in C^{I}\right\}\\ & {\left(\forall R.C\right)}^{I}=\left\{x|\forall y.\left(x,y\right)\in R^{I}\wedge y\in C^{I}\right\} \end{array}$$

#### Definition 2.4 (Simple-Form Concept)

A concept is said to be in simple form, if the maximum level of nested quantifiers in this concept is less than 2.

For example, given an atomic concept *A* , both *A* and ∃*R.A* are simple-form concepts, while ∃*R*_1_.(*A*∃*R*_2_.*A*) is not, since its maximum level of nested quantifiers is two. Notice however, an arbitrary concept can be *linearly* reduced to the simple form by assigning new concept names for fillers of the quantifiers. For example, ∃*R*_1_.∃*R*_2_.*C* can be converted to ∃*R*_1_.*D* by letting *D* ≡ ∃*R*_2_.*C* where *D* is a new concept name.

##### Assumption

For accuracy of the technique presented in this paper, without loss of generality, we assume all ontology concepts are in simple form as defined previously, and the concept in any concept assertion is atomic.

### 2.2. DL Ontologies and Reasoning

#### Definition 2.5 (SHIO Ontology)

*A* SHIO *ontology is a tuple, denoted* K=(T,A), *where* T *is called a TBox and* A *is called an ABox.*

The TBox T is constituted by a finite set of role inclusion axioms (*i.e.* *R*_1_ ⊑ *R*_2_ with *R*_1_, *R*_2_ ∈ R*) and a finite set of concept inclusion axioms in the form of *C* ⊑ *D* and *C* ≡ *D* , where *C* , *D* are SHI concepts. The former is called a *general concept inclusion axiom* (GCI), and the latter can be simply converted into two GCIs as *C* ⊑ *D* and *D* ⊑ *C* .

The ABox A consists of a finite set of assertions, in the form of *A*(*a*) (concept assertion) and *R*(*a,b*) (role assertion), where *A* is a concept, *R* is a role, and *a*, *b* are named individuals in *I* . In a role assertion *R*(*a,b*) , individual *a* is referred to as a *R -predecessor* of *b* , and *b* is a *R -successor* (or *R*^−^ *-predecessor*) of *a* . If *b* is a *R* -successor of *a* , *b* is also called a *R -neighbor* of *a* .

An interpretation I satisfies an axiom *C* ⊑ *D* (written I⊨C⊑D), iff $C^{I}\subseteq D^{I}$ , and I satisfies an axiom or assertion:

$$\begin{array}{cc} & R_{1}⊑R_{2}\;\mathrm{iff}\;R_{1}^{I}\subseteq R_{2}^{I}\\ & C\left(a\right)\;\mathrm{iff}\;a^{I}\in C^{I}\\ & R\left(a,b\right)\;\mathrm{iff}\;\left(a^{I},b^{I}\right)\in R^{I} \end{array}$$

If I satisfies every axiom and assertion of an ontology K , I is called a *model* of K , written I⊨K . In turn, K is said *satisfiable* iff it has at least one model; otherwise, it is *unsatisfiable* or *inconsistent*.

#### Definition 2.6 (Logical Entailment)

*Given an ontology* K and an axiom α , α is called a logical entailment of K, *denoted* K⊨α, *if α is satisfied in every model of* K.

#### Definition 2.7 (Instance Checking)

*Given an ontology* K, a DL concept C and an individual a ∈ I , instance checking is defined to test if K⊨C(a) *holds.*

Notice that, instance checking is considered the central reasoning service for information retrieval from ontology ABoxes [19], and more complex reasoning services, such as instance retrieval, can be realized based on this basic service. Instance checking can also be viewed as a procedure of individual “*classification*” that verifies if an individual can be classified into some defined DL concepts. An intuition to implement this instance checking service is to convert it into a concept subsumption test by using the so-called *most specific concept* (MSC) method.

#### Definition 2.8 (Most Specific Concept [20])

*Let* K=(T,A) be an ontology, and a be an individual in I . A concept C is called the most specific concept for a w.r.t. A, *written* MSC(A,a), *if for every concept D that* K⊨D(a), I⊨C⊑D.

The MSC method turns the instance checking into a TBox reasoning problem. That is, once the most specific concept MSC(A,a) of an individual *a* is known, to decide if K⊨D(a) holds for an arbitrary concept *D* , it suffices to test if T⊨MSC(A,a)⊑D [10].

Ontology reasoning algorithm in current systems (e.g. Pellet, and HermiT, etc.) are based on (hyper) tableau algorithms [4] [6] [7] [21]. For details of a standard tableau algorithm for *SHIO* , we refer readers to the work in [22].

## 3. Classification of Individuals

The MSC method for individual checking is based on the idea that, an individual can be classified into a given concept *D* , if and only if there exists a concept behind its ABox assertions subsumed by *D* [17] [18] [20]. Computation of the MSC for a given individual then demands converting its ABox assertions into a concept. This task can be easily accomplished if the individual possesses only concept assertions, by simply collapsing the involved concepts into a single term using the concept conjunction. When role assertions are involved, however, a more complex procedure is demanded, and the method we used here is called *rolling-up* [23], which is elaborated in the next section.

Using the MSC method for instance checking might eliminate the memory limitation for reasoning with large ABoxes, especially when the ABox A consists of data in great diversity and isolation. This is simply because each computed MSC(A,a) should comprise of only related information of the given individual, and makes the subsumption test (*i.e.* T⊨MSC(A,a)⊑D) as efficient as an ontology reasoning that explores only a (small) portion of A .

However, as discussed in Section 1, due to the support of existential restrictions in DLs, great complexity for computation of MSC's may arise when role assertions are involved. Besides, due to the *completeness* that should be guaranteed by each MSC(A,a) *i.e.* the MSC should be subsumed by any concept that the individual *a* belongs to.), the resulting MSC's may turn out to be a very large concept, whenever there is a great number of individuals in A connected to each other by role assertions. In the *worst* case, reasoning with a MSC may degenerate into a complete ABox reasoning that could be prohibitively expensive. For example, when MSC(A,a) preserves complete information of A , its interpretation will form a tableau, the size of which can be in the same scale of A .

### 3.1. The Call-by-Need Strategy

Since the larger than desired sizes of MSCs are usually caused by its completeness as discussed above, a possible optimization to the MSC method is thus to abandon the completeness that is required to deal with any query concepts, and to apply a “*call-by-need*” strategy. That is, for an arbitrary query concept *D* , instead of computing the MSC for each individual *a* , we compute a concept that is only *specific-enough* to determine if *a* can be classified into *D* . As suggested by its name, this revision to the original MSC method, instead of taking the complete information of individual *a* , when computing the “MSC”, will consider only the ABox assertions that are relevant to the current query concept.

A simple way to realize this strategy is to assign a fresh name *A* every time to a given (complex) query concept *D* by adding the axiom *A* ≡ *D* to $T^{1}$^1^, and to concentrate only on ABox assertions that would (probably) classify an individual *a* into *A* w.r.t. T . Consequently, this implementation requires an analysis of the ontology axioms/assertions, such that the possibility of each role assertion to affect individual classification (w.r.t. named concept in T ) can be figured out. Computation of a specific-enough concept should then concentrate on role assertions that are *not impossible*. We abuse the notation here to denote this specifi-cenough concept for individual *a* w.r.t. ABox A , current query concept Q , and named concepts in T as ${\mathrm{MSC}}_{T}\left(A,Q,a\right)$ , and we call the method that uses ${\mathrm{MSC}}_{T}$ for instance checking the ${\mathrm{MSC}}_{T}$ method.

### Definition 3.1

*Let* K=(T,A) *be an ontology, a be an individual in* A, *and* Q a current query concept for individuals. A concept C is called a specific-enough concept for a w.r.t. named concepts in T , Q *and* A, *written* ${\mathrm{MSC}}_{T}\left(A,Q,a\right)$, *if* K⊨Q(a), T⊨C⊑Q .

Since in our procedure we will add the query concept Q into T as a named concept, we can simplify the notation ${\mathrm{MSC}}_{T}\left(A,Q,a\right)$ as ${\mathrm{MSC}}_{T}\left(A,a\right)$ .

### 3.2. A Syntactic Premise

To decide whether a role assertion could affect classification of a given individual, a sufficient and necessary condition as stated previously is that, the concept behind this assertion conjuncted with other essential information of the individual should be subsumed by the given concept w.r.t. T [17] [18] [20]. Formally, for a role assertion *R*(*a,b*) that makes individual *a* classified into a concept *A* , the above sufficient and necessary condition in SHI can be expressed as:

(1) $$K⊨\exists R.B⊓A_{0}⊑A$$

where *b* ∈ *B* is entailed by K , and concept *A*_0_ summarizes the rest of the information of *a* that is also essential to this classification, with *A*_0_ ⊑ *A* .

As shown in [16], for subsumption (1) to hold when *A* is a named concept, there must exist some role restriction ∃*R′.C* with *R* ⊑ *R*′ in left-hand side of TBox axioms (see (2) and the following axiom equivalency) for concept definition; otherwise ∃*R.B* is not comparable (w.r.t. subsumption) with other concepts (except Τ and its equivalents). This syntactic condition for the deduction of (1) is formally expressed in the following proposition.

#### Proposition 3.1 ([16])

*Let* K=(T,A) *be a* SHI *ontology with simple-form concepts only*, ∃*R.B, A*_0_ *and A be* SHI *concepts, where A is named. If*

$$K⊨\exists R.B⊓A_{0}⊑A$$

with *A*_0_ ⋢ *A* , there must exist some GCIs in T in the form of:

(2) $$\exists R^{\prime }.C_{1}⋈C_{2}⊑C_{3}$$

where *R* ⊑ *R*′ and ⋈ is a place holder for ⊔ and ⊓ , *C_i_*'s are SHI concepts. Also note the following equivalence:

$$\begin{array}{cc} & \exists R.C⊑D\equiv \neg D⊑\forall R.\neg C\\ & \exists R.C⊑D\equiv C⊑\forall R^{-}.D\\ & C_{1}⊓C_{2}⊑D\equiv C_{1}⊑D⊔\neg C_{2} \end{array}$$

This proposition is proven in [16]. It states in fact a syntactic premise in SHI for a role assertion to be essential for some individual classification. That is, if a role assertion *R*(*a, b*) is essential for derivation of *A*(*a*) for some named concept *A* , there must exist a related axiom in T in the form of (2) for *R* ⊑ *R*′ . We denote this syntactic premise for *R*(*a, b*) to affect *a* 's classification as SYN_COND . Using this condition, we can easily rule out role assertions that are definitely irrelevant to the query concept and will not be considered during the computation of a ${\mathrm{MSC}}_{T}$ .

## 4. Computation of MSC_T_

In this section, we present the technique that computes a ${\mathrm{MSC}}_{T}$ for a given individual w.r.t. a given query. We assume the ABox considered here is consistent, since for any inconsistent ABox, the ${\mathrm{MSC}}_{T}$ is always the bottom concept ⊥ [24]. Essentially, the task is to convert ABox assertions into a single concept for a given individual, using the concept conjunction and the so-called *rolling-up* technique. This rolling-up technique was introduced in [23] to convert conjunctive queries into concept terms, and was also used by [25] to transform datalog rules into DL axioms. We adapt this technique here to roll up ABox assertions into DL concepts.

### 4.1. The Rolling-Up Procedure

Converting concept assertions into a concept is straightforward by simply taking conjunction of the involved concepts. When role assertions are involved, the rolling-up technique can be used to transform assertions into a concept by eliminating individuals in them. For example, given the following assertions

(3) Male(Tom),has Parent(Tom,Mary),Lawyer(Mary),

transforming them for individual Tom using the rolling up and concept conjunction can generate a single concept assertion

(Male⊓∃has Parent.Lawyer)(Tom)

#### Generalize the Information

The transformation here is for individual Tom, and if individual Mary is not explicitly indicated in the query, it should be sufficient to rewrite has Parent (Tom,Mary), Lawyer (Mary) into ∃has Parent.Lawyer (Tom) , without loss of any information that is essential for query answering. Even if Mary is explicitly indicated in the query, we can still eliminate it by using a *representative* concept that stands for this particular individual in the given ABox [26]. For example, we can add an assertion *A*_mary_ (Mary) to the ABox, where *A*_mary_ is a new concept name and a representative concept for individual Mary. The above role assertions for Tom then can be transformed into concept ∃has Parent.(Lawyer ⊓ *A*_mary_)(Tom)(Mary) ; and if the query is also rewritten using concept Mary, the *completeness* of the query answering can be guaranteed, as indicated by the following theorem [26].

#### Theorem 4.1 ([26])

*Let* K=(T,A) *be a DL ontology, a, b be two individuals in* A , *R a role, and C*_1_, ..., C_n_ DL concepts. Given a representative concept name A_b_ for b not occurring in K :

$$K⊨R\left(a,b\right)\wedge C_{1}\left(b\right)\wedge \cdots \wedge C_{n}\left(b\right)$$

if and only if

$$K\cup \left\{A_{b}\left(b\right)\right\}⊨\exists R.\left(A_{b}⊓C_{1}⊓\cdots ⊓C_{n}\right)\left(a\right)$$

The rolling-up procedure here can be better understood by considering a *graph* induced by the role assertions to be rolled up, which is defined as follows:

#### Definition 4.1

*A set of ABox role assertions in* SHI *can be represented by a graph* G, in which there is a node x for each individual x in the assertions, and an edge between node x and y for each role assertion R( x, y) .

Notice that, due to the support of inverse roles in SHI , edges in G are not directed. A *role path* in the graph G is then defined as a set of roles corresponding to the set of edges (no duplicate allowed) leading from one node to another. For example, given assertions *R*_1_(*x, y*) and *R*_2_(*x, y*), the role path from *x* to *z* is $\left\{R_{1},R_{2}^{-}\right\}$, and its reverse from *z* to *x* is $\left\{R_{2},R_{1}^{-}\right\}$.

The rolling-up for a given individual *x* is then able to generate concepts by eliminating individuals in branches of the *tree-shaped* graph G , starting from the leaf nodes and rolling up towards the root node indicated by *x* . Moreover, all the information of each individual being rolled up should be absorbed into a single concept by conjunction during the procedure. For example, if we have additional assertions

has Sister(Mary,Ana)andProfessor(Ana)

for Mary in (3), the rolling-up for Tom should then generate concept

Male⊓has Parent.(Lawyer⊓has Sister.Professor)

##### Inverse Role

The support of inverse roles in SHI makes this rolling-up procedure bidirectional, thus, making it applicable to computing ${\mathrm{MSC}}_{T}$ for any individual in the ABox. For example, to compute a ${\mathrm{MSC}}_{T}$ for individual Mary in example (3), we simply treat this individual as the root, and roll up assertions from leaves to root to generate the concept

$$\text{Lawyer}⊓{\text{has Parent}}^{-}.\text{Male}$$

##### Transitive Role

In the rolling-up procedure, no particular care needs to be taken to deal with transitive roles, since any role assertions derived from transitive roles will be automatically preserved [26]. For example, given *R* a transitive role, *R*(*a,b*) , *R*(*b,c*) two role assertions, and *B*(*b*), *C*(*c*) two concept assertions in the ABox, rolling-up these four assertions for individual *a* can generate assertion ∃*R*.(*B* ⊓ ∃*R.C*)(*a*) , from which together with the TBox axioms, we can still derive the fact that

(∃R.(B⊓∃R.C)⊓∃R.C)(a)

##### Assertion Cycles

This rolling-up technique, however, may suffer information loss if the graph G contains cycles (*i.e.* a role path leading from one node to itself without duplicate graph edges). For example, given the following two assertions:

$$R_{1}\left(x,y\right)\;\text{and}\;R_{2}\left(x,y\right)$$

individuals *x* and *y* are related by two roles, and a cycle is thus induced in the corresponding graph. Rolling-up assertions for individual *x* using the method described above might generate concept $\exists R_{1}.T⊓\exists R_{2}.T$ , and the fact that *x* is connected to the same individual *y* through different roles is lost. Consequently, this may compromise the resulting concept as a specific-enough concept for *x* to answer the current query. For example, let *C* be a query concept defined as:

$$\exists R_{1}.\exists R_{2}^{-}.\exists R_{1}.T$$

It can be found out through ABox reasoning that individual *x* is an instance of *C* ; while on the other hand, it is also not difficult to figure out that $\exists R_{1}.T⊓\exists R_{2}.T$ is not subsumed by *C* .

Multiple solutions to this problem have been proposed, such as an approximation developed by [27], and the use of cyclic concept definition with greatest fixpoint semantics [24] [28]. In this paper, we choose to use the *nominal* (e.g. {*x*}) to handle circles as suggested by [19] [20], which allows explicit indication of named individuals in a concept, hence, being able to indicate the joint node of a cycle. The above two assertions in (4) then can be transformed into a concept for individual *x* as either $\exists R_{1}.\left\{y\right\}⊓\exists R_{2}.\left\{y\right\}$ or $\left\{x\right\}⊓\exists R_{1}.\exists R_{2}^{-}.\left\{x\right\}$, each with the nominal used for a chosen joint node, and both preserve complete information of the cycle. In our approach, when rolling up a cycle in G , we always treat the cycle as a single branch and generate concepts of the second style. That is, our procedure will treat a chosen joint node *x* as both the tail (*i.e.* leaf) and the head (*i.e.* root) of the branch. For clarity of the following presentation, we denote the tail as *x^t^* and the head as *x^h^* .

Based on the discussion so far, the transformation of assertions for a given individual now can be formalized as follows. Let *x* be a named individual, and *γ* be an ABox assertion for *x* . *γ* can be transformed into a concept *C_γ_* for *x* :

Cγ={Cifγ=C(x)∣C(xh)∃R.Difγ=R(x,y),andD(y)∃R−.Difγ=R(y,x),andD(y){xh}⊓∃R.{xt}ifγ=R(xh,xt){xh}⊓∃R.Difγ=R(xh,y),andD(y){xh}⊓∃R−.Difγ=R(y,xh),andD(y){xt}ifγ=R(xt,y)∣R(y,xt)∣C(xt)}

Notice that, concept *D* here is a obtained concept when rolling up branch(es) in G up to node *y* , and transforming any assertion of a cycle tail *x^t^* always generates {*x^t^*}, as complete information of *x* will be preserved when rolling up to the head *x^h^* . Thereafter, given a set *S* of all assertions of individual *x* , ${\mathrm{MSC}}_{T}\left(A,x\right)$ can be obtained by rolling up all branches induced by role assertions in *S* and taking the conjunction of all obtained concepts. When *S* is empty, however, individual *x* in the ABox can only be interpreted as an element of the entire domain, and thus, the resulting concept is simply the top entity T . Computation of a ${\mathrm{MSC}}_{T}\left(A,x\right)$ then can be formalized using the following equation:

MSCT(A,x)={TifS=∅⊓γ∈SCγif otherwise}

### 4.2. Branch Pruning

To apply the call-by-need strategy, the previously defined syntactic premise SYN_COND is employed, and a branch to be rolled up in graph G will be truncated at the point where the edge does not have SYN_COND satisfied. More precisely, if an assertion *R*(*x, y*) in a branch does not have the corresponding SYN_COND satisfied, it will not affect any classification of individual *x* w.r.t. T . Moreover, any effects of the following assertions down the branch will not be able to propagate through *R*(*x, y*) to *x* , and thus should not be considered during the rolling-up of this branch.

This branch pruning technique could be a simple yet an efficient way to reduce complexity of a ${\mathrm{MSC}}_{T}$ , especially for those practical ontologies, where many of the ABox individuals may have a huge number of role assertions and only a (small) portion of them have SYN_COND satisfied. For a simple example, consider an individual *x* in an ontology ABox with the following assertions:

$$R_{1}\left(x,y_{1}\right),R_{2}\left(x,y_{2}\right),\cdots ,R_{n}\left(x,y_{n}\right)$$

where *n* could be a very large number and only *R*_1_ has SYN_COND satisfied. Rolling up these assertions for individual *x* without the pruning will generate the concept

$$\exists R_{1}.C_{1}⊓\exists R_{2}.C_{2}⊓\cdots ⊓\exists .R_{n}.C_{n}$$

where *y_i_* ∈ *C_i_* . Using this concept for any instance checking of *x* could be expensive, as its interpretation might completely restore the tableau structure that is induced by these assertions. However, when the pruning is applied, the new ${\mathrm{MSC}}_{T}$ should be ∃*R*_1_.*C*_1_, the only role restriction that is possible to affect individual classification of *x* w.r.t. named concepts in T .

Going beyond such simple ontologies, this optimization technique may also work in complex ontologies, where most of the role assertions in ABox could have SYN_COND satisfied. For example, consider the following assertions

$$R_{0}\left(x_{0},x_{1}\right),R_{1}\left(x_{1},x_{2}\right),\cdots ,R_{n}\left(x_{n},x_{n+1}\right)$$

with all roles except *R*_2_ having SYN_COND satisfied. Rolling up these assertions for individual *x*_0_ will start from the leaf *x*_*n*+1_ up towards the root *x*_0_ , and generate the concept

$$\exists R_{0}.\exists R_{1}.\cdots .\exists R_{n}.C$$

where *x*_*n*+1_ ∈ *C* . However, with pruning applied, the rolling-up in this branch will start from *x*_2_ instead of *x*_*n*+1_ , since *R*_2_(*x*_2_, *x*_3_) will not affect classification of individual *x*_2_ w.r.t. T and the branch is truncated at this point.

Furthermore, with branch pruning, cycles should only be considered in the truncated graph, which may further simplify the computation of ${\mathrm{MSC}}_{T}$'s.

### 4.3. Further Optimization and Implementation

The branch pruning here is based on SYN_COND to rule out irrelevant assertions, which in fact can be further improved by developing a more rigorous premise for a role assertion to affect individual classification. For exposition, consider the following ontology K :

({∃R.C⊑D},{¬D(a),R(a,b),¬C(b)})

When computing ${\mathrm{MSC}}_{T}{\left(A,a\right)}_{T}\left(A,a\right)$ using the proposed method, assertion *R*(*a,b*) will be rolled up as the corresponding SYN_COND is satisfied. However, it is not difficult to see that, *R*(*a,b*) here actually makes no contribution to *a* ’s classification, since individual *b* is in the complement of concept *C* , making *a* an instance of ∃R.¬C . Besides, individual *a* has already been asserted as an instance of concept ¬D , and hence cannot be classified into *D* unless the ABox is inconsistent.

With these observations, a more rigorous premise based on SYN_COND can be derived. That is, to determine the possibility for *R*(*a,b*) to affect classification of individual *a* , beyond checking in T the existence of any axiom in the form of

$$\exists R^{\prime }.C_{1}⋈C_{2}⊑C_{3}$$

with *R* ⊑ *R*′ and ⋈ a place holder for ⊔ and ⊓ , we also check the following cases for any found axiom:

#### Case 1

If there is any concept *B*_0_ in explicit concept assertions of individual *b* , such that $K⊨B_{0}⊑\neg C_{1}$ ,

#### Case 2

If there is any concept *A*_0_ in explicit concept assertions of individual *a* , such that $K⊨A_{0}⊑\neg {\left(C_{3}⊔\neg C_{2}\right)}^{2}$^2^ or $K⊨A_{0}⊑\neg C_{3}$ , respectively for ⋈ standing for ⊓ or ⊔ .

If either one of the above cases happens, that particular ∃*R*′.*C*_1_ in the left hand side of the axiom in fact makes no contribution to the inference of *a*'s classification, unless the ABox is *inconsistent* where MSCs are always ⊥ [24]. Thus, a revised condition requires not only the existence of a related axiom in the form of (2) but also with none of the above cases happening. We denote this condition as SYN_COND* , and use it to rule out assertions that are irrelevant to the current query.

This optimization is useful to prevent rolling-up of role assertions in an arbitrary direction on existence of related axioms in T . Instead, it limits the procedure to the direction that is desired by the original intention underlying the design of the given ontology. For example, in (5), the axiom ∃*R.C* ⊑ *D* specifies that, any individual having a *R* -neighbor in *C* is an instance of *D* and any individual having a *R*^−^ -neighbor in ¬D is an instance of $\neg C^{3}$^3^. However, if individual *x* is asserted to have a *R* -neighbor in ¬C or a *R*^-^ -neighbor in *D* , that role assertion should not be rolled-up for *x* just on existence of this axiom.

**Figure 2:**
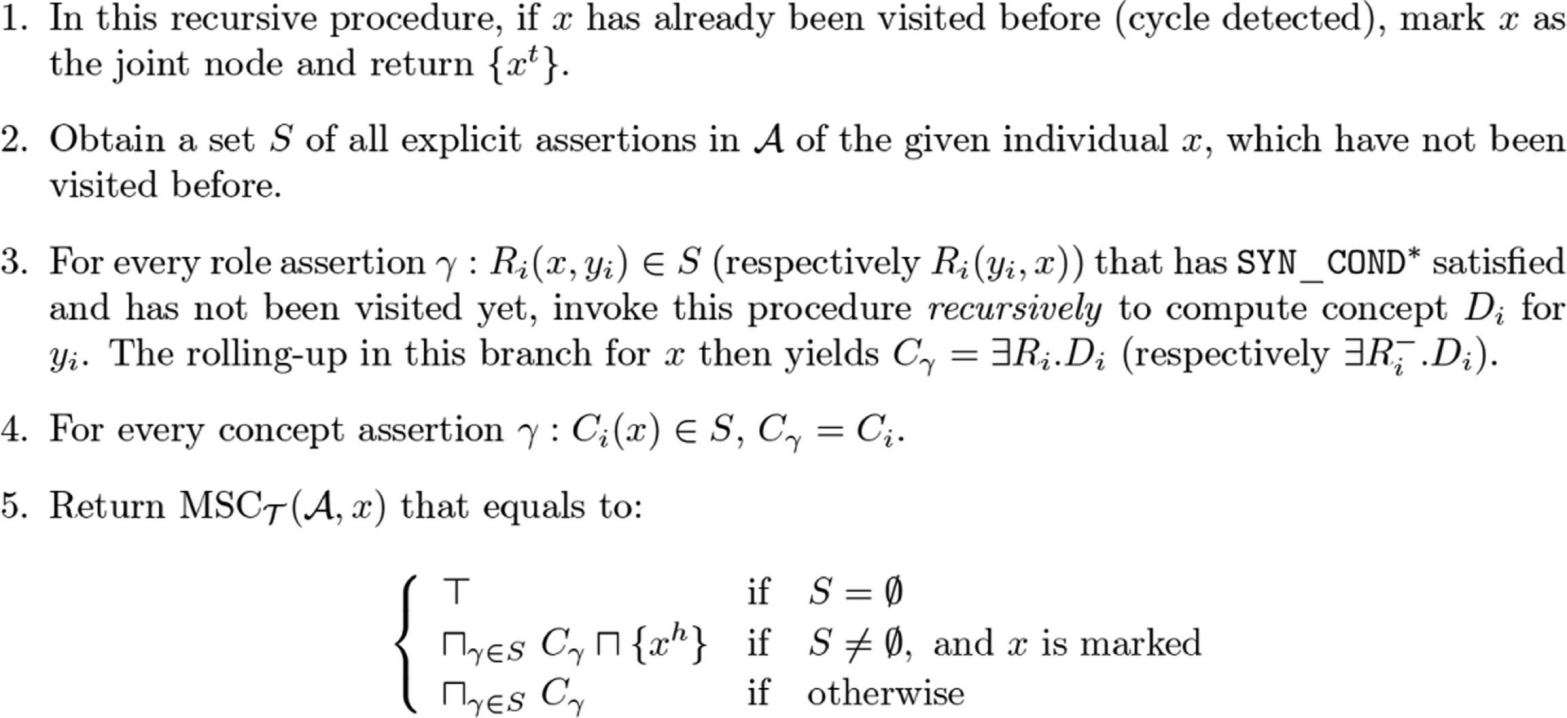
A recursive procedure for computation of ${\mathrm{MSC}}_{T}\left(A,x\right)$ .

With all the insights discussed so far, an algorithm for computation of ${\mathrm{MSC}}_{T}\left(A,a\right)$ is presented here as a *recursive* procedure, the steps of which are summarized in **Figure 2**.

##### Proposition 4.1 (Algorithm Correctness)

*The algorithm presented in* **Figure 2** *computes a* ${\mathrm{MSC}}_{T}\left(A,a\right)$ *for a given* SHI *ontology* (T,A) *and an individual* *x* in A .

###### Proof

We prove by induction.

####### Basis

For a leaf node *x* in G , which has no other role assertions except those up the branches, rolling it up yields the conjunction of concepts in its concept assertions, which preserves sufficient information of the part of the branch being rolled so far. If *x* is the tail of a cycle, returning {*x^t^*} is sufficient, as other information of *x* will be gathered when the rolling-up comes to the head.

####### Inductive Step

Let *x* be a node in the middle of some branch(es) in G . For every role assertion *R_i_*(*x,y_i_*) of *x* down the branch, assume the procedure generates a concept *D_i_* for rolling up to each node *y_i_*, which preserves sufficient information (w.r.t. current query) of the part of branches being rolled up so far. Then, rolling up each *R_i_*(*x, y_i_*) generates ∃*R.D_i_*, and together with concept assertions of *x*, the concept conjunction preserves sufficient information of all branches being rolled up to *x* . If *x* is marked as a joint node of a cycle, {*x^h^*} is also in the conjunction, so that the circular path property can be preserved. If *x* is the root node, the conjunction is thus a ${\mathrm{MSC}}_{T}\left(A,x\right)$ that preserves sufficient information of *x* w.r.t. current query.

This algorithm visit every relevant ABox assertion at most once, and it terminates after all related assertions are visited.

## 5. Related Work

The idea of most specific concept for instance checking was first discussed in [18], and later extensively studied by [17] [20] for the algorithms and the computational complexity. To deal with existential restrictions when computing the most specific concept, [24] [28] [29] discussed the use of cyclic concepts with greatest fixpoint semantics for preservation of information induced by the role assertions, and [27] also proposed an approximation for most specific concept in DLs with existential restrictions.

On the other hand, for efficient ABox reasoning and instance checking, various optimization techniques have been developed, including lazy unfolding, absorption, heuristic guided search, exploration of Horn clauses of DLs [4] [5] [22], model merging [2] and extended pseudo model merging technique [3] [30].

A common direction of these optimization techniques is to reduce the high degree of nondeterminism that is mainly introduced by GCIs in the TBox: given an GCI *C* ⊑ *D* , it can be converted to a disjunction C⊔¬D , for which a tableau algorithm will have to nondeterministically choose one of the disjuncts for tableau expansion, resulting in an exponential-time behavior of the tableau algorithm w.r.t. the data size. Absorption optimizations [22] [31] [32] were developed to reduce such nondeterminism by combining GCIs for unfoldable concepts, such that the effectiveness of lazy unfolding can be maximized. For example, axioms *A* ⊑ *C* and *A* ⊑ *D* can be combined into *A* ⊑ *C* ⊓ *D*, where *A* is a named concept; then the inference engine can deduce *C* ⊓ *D* (*a*) if the ABox contains *A* (*a*). Notice however, this technique may allow only parts of TBox axioms to be absorbed, thus, may not be able to eliminate all sources of nondeterminism especially when ontologies are complex. Based on the absorption optimization, [33] proposed an approach for efficient ABox reasoning for ALCIQ that will convert ABox assertions into TBox axioms, apply a absorption technique on the TBox, and covert instance retrieval into concept satisfaction problems.

Another way to reduce nondeterminism is the exploration of Horn clauses in DLs, since there exist reasoning techniques for Horn clauses that can be deterministic [5] [34]. [5] takes advantage of this in their HermiT reasoner by preprocessing a DL ontology into DL-clauses and invoking the hyperresolution for the Horn clauses, avoiding unnecessary nondeterministic handling of Horn problems in existing DL tableau calculi.

For non-Horn DL, techniques such as model merging [2] and pseudo model merging [30] can be used to capture some deterministic information of named individuals. These techniques are based on the assumption of a consistent ABox and the observation that usually individuals are members of a small number of concepts. The (pseudo) model merging technique merges clash-free tableau models that are constructed by disjunction rules for a consistent ABox, and can figure out individuals that are obviously non-instance of a given concept. For example, if in one tableau model individual *a* belongs to concept *C* while in another *a* belongs to ¬C , it is then obvious that individual *a* cannot be deterministically inferred to be an instance of concept *C* , thus, eliminating the unnecessary instance checking for *C*(*a*) .

Another option for scalable ABox reasoning is the use of tractable DL languages. For example, the description logic *EL* and its extension *EL*^++^ , which allow existential restrictions and conjunction as introduced by [35] [36], possess intriguing algorithmic properties such that the satisfiability problem and implication in this DL language can be determined in polynomial time. Another notable example of lightweight DLs is the socalled *DL-LITE* family identified by [37], which is specifically tailored to capture basic DL properties and expressivity while still be able to achieve low computational complexity for both TBox and ABox reasoning. In [9] [38] they further identified that, for conjunctive queries that are FOL-reducible, answering them in ontologies of any DL-LITE logic enjoys a LOGSPACE data complexity.

Based on the above lightweight DLs, efficient DL reasoners are developed, such as OWLIM [39], ELK reasoner [40], and Oracle's native inference engine for RDF data sets [41].

[42] proposed an approximation technique for instance retrieval, which computes both lower bound and upper bound of an answer set of individuals for a given query concept. Their approach invokes an axiom rewriting procedure that converts an ontology in Horn DL into a datalog program, and then uses Oracle's native inference engine to derive the bounds for query answering.

Recently, techniques for partitioning or modularizing ABoxes into logically-independent fragments have been developed [15] [16]. These techniques partition ABoxes into logically-independent modules, such that each will preserve complete information of a given set of individuals, and thus can be reasoned independently w.r.t. the TBox and be able to take advantage of existing parallel-processing techniques.

## 6. Empirical Evaluation

We implemented the rolling-up procedures for computation of ${\mathrm{MSC}}_{T}$'s based on the OWL API^4^, and evaluated the MSC method for instance checking and retrieving on a lab PC with Intel(R) Xeon(R) 3.07 GHz CPU, Windows 7, and 1.5 GB Java Heap. For the test suite, we have collected a set of well-known ontologies with large ABoxes:

1) LUBM(s) (LM) are benchmark ontologies generated using the tool provided by [43];
2) Arabidopsis thaliana (AT) and Caenorhabditis elegans (CE) are two biomedical ontologies^5^, sharing a common TBox called *Biopax* that models biological pathways;
3) DBpedia* (DP) ontologies are *extended* from the original DBpedia ontology [44]: expressivity of their TBox is extended from ALF to SHI by adding complex roles and concepts defined on role restrictions; their ABoxes are obtained by random sampling on the original triple store.

Details of these ontologies can be found in **Table 1**, in terms of DL expressivity, number of atomic concepts (# Cpts), TBox axioms (# Axms), named individuals (# Ind.), and ABox assertions (# Ast.). Notice that, DL expressivity of AT and CE is originally SHIN , but in our experiments, number restrictions (*i.e.* N) in their ontology TBox are removed.

### 6.1. Complexity of ${\mathrm{MSC}}_{T}$

Using the MSC (or ${\mathrm{MSC}}_{T}$) method, the original instance checking problem is converted to a subsumption test, the complexity of which could be computationally high w.r.t. both size of a TBox and size of the testing concepts [10]. Therefore, when evaluating the rolling-up procedure for computation of ${\mathrm{MSC}}_{T}$'s, one of the most important criteria is the size of each resultant ${\mathrm{MSC}}_{T}$, as it is the major factor to the time efficiency of a subsumption test, given a relatively static ontology TBox.

As we already know, one of the major source of complexity in ontology reasoning is the so-called “*and-branching*”, which introduces new individuals in the tableau expansion through the ∃-rule, and affects the searching space of the reasoning algorithm as discussed in [10]. Thus, when evaluating sizes of computed ${\mathrm{MSC}}_{T}$'s, we measure both the level of nested quantifiers (*i.e.* quantification depth) and the number of conjuncts of each ${\mathrm{MSC}}_{T}$. For example, the concept

$$\exists R_{1}.C_{1}⊓\exists R_{2}.\left(C_{2}⊓\exists R_{3}.C_{3}\right)$$

has quantification depth 2 and number of conjuncts 2 (*i.e.* and $\exists R_{2}.\left(C_{2}⊓\exists R_{3}.C_{3}\right)$).

#### 6.1.1. Experiment Setup

To evaluate and show efficacy of the proposed strategy and optimization, we have implemented the following three versions of the rolling-up method for comparison:

**V1** The original rolling-up procedure adapted to ABox assertions without applying the call-by-need strategy, which computes the most specific concept w.r.t. A for a given individual;
**V2** The rolling-up procedure with the proposed *call-by-need* strategy based on SYN_COND , which features the branch pruning as fully discussed in Section 4.2;
**V3** The rolling-up procedure with the *call-by-need* strategy based on SYN_COND* discussed in Section 4.3.

**Figure 3:**
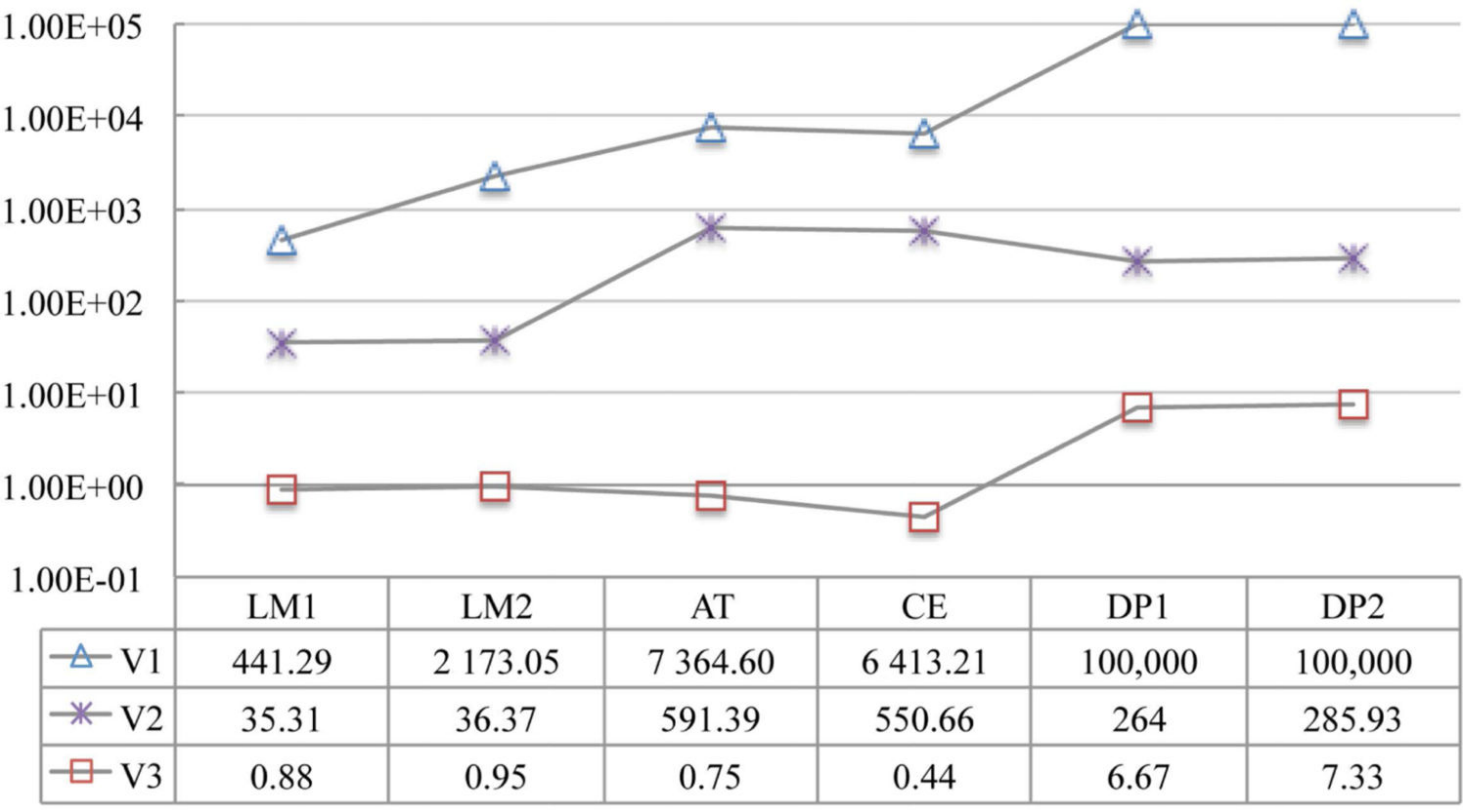
Average time (ms) on computation of a ${\mathrm{MSC}}_{T}$ . Timeout is set to be 100,000 ms.

We compute the ${\mathrm{MSC}}_{T}$ for each individual in every ontology using the three methods respectively, and report in **Table 2** and **Table 3** the maximum and the average of quantification depth and number of conjuncts of the concepts, respectively. We also demonstrate the running-time efficiency of the optimized rolling-up procedure by showing the average time spent on computation of a ${\mathrm{MSC}}_{T}$ for each individual in **Figure 3**.

#### 6.1.2. Result Analysis

As we can see from **Table 2** and **Table 3**, the sizes of ${\mathrm{MSC}}_{T}$'s generated by V2 and V3 are significantly smaller than those generated by V1 (the original method), which are almost in the same scale of size of the corresponding ontology ABox. The large size of ${\mathrm{MSC}}_{T}$'s from V1 is caused by the fact that most individuals (greater than 99%) in each of these ontologies are connected together by role paths in the graph. The bulk of each ${\mathrm{MSC}}_{T}$ makes the original MSC method completely inefficient and unscalable for answering object queries, as a subsumption test based on these concepts would be prohibitively expensive as a complete ABox reasoning. Thus, the comparison here reflects the potential and the importance of our proposed optimizations in this paper, which revive the MSC (*i.e.* ${\mathrm{MSC}}_{T}$ method as an efficient way for instance checking and object query answering.

The comparison between V2 and V3 demonstrates the efficacy of the optimization technique discussed in Section 4.3, which could prevent the rolling-up in arbitrary directions by providing a more rigorous precondition based on SYN_COND . This optimization could be useful in many practical ontologies, especially when their ABoxes contain “hot-spots” individuals that connect (tens of) thousands of individuals together and could cause the rolling-up to generate concepts with a prohibitive quantification depth.

In particular, in our previous study of modularization for ontology ABoxes [16], the biomedical ontologies (*i.e.* AT and CE ) are found to be complex with many of their ontology roles (33 out of 55) used for concept definitions, and their ABoxes are hard to be modularized even with various optimization techniques applied [16]. However, in this paper, we found much simpler ${\mathrm{MSC}}_{T}$'s can also be achieved in these complex ontologies when the optimization (*i.e.* SYN_COND*) is applied. For example, the maximum quantification depths of computed ${\mathrm{MSC}}_{T}$'s in both AT and CE are decreased significantly from more than 1000 to less than 10. Nevertheless, it should also be noted that, effectiveness of this optimization may vary on different ontologies, depending on their different levels of complexity and different amount of explicit information in their ABoxes that can be explored for optimization.

### 6.2. Reasoning with ${\mathrm{MSC}}_{T}$

In this section, we will show the efficiency that can be achieved when using the computed ${\mathrm{MSC}}_{T}$ for instance checking and retrieving. We conduct the experiments on the collected ontologies, and measure the average reasoning time that is required when performing instance checking (for every ABox individual) and instance retrieval using the ${\mathrm{MSC}}_{T}$ method, respectively.

#### 6.2.1. Experiment Setup

We will not compare our method with a particular optimization technique for ABox reasoning, such as lazy unfolding, absorption, or model merging, etc., since they have already been built into existing reasoners and it is usually hard to control reasoners to switch on or off a particular optimization technique. Additionally, the ${\mathrm{MSC}}_{T}$ method still relies on the reasoning services provided by the state-of-art reasoners. Nevertheless, we do compare the reasoning efficiency between the ${\mathrm{MSC}}_{T}$ method and a regular complete ABox reasoning using existing reasoners, but only to show the effectiveness of the proposed ${\mathrm{MSC}}_{T}$ method for efficient instance checking and data retrieving. Moreover, we also compare the ${\mathrm{MSC}}_{T}$ method with the ABox partitioning method (*modular reasoning*) developed in [16], as they are developed based on the similar principles and both allow parallel or distributed reasoning.

The ${\mathrm{MSC}}_{T}$'s here are computed using algorithm V3, and the ABox partitioning technique used is the most optimized one presented in [16]. For a regular complete ABox reasoning, the reasoners used are OWL DL reasoners, HermiT [5] and Pellet [6], each of which has its particular optimization techniques implemented for the reasoning algorithm. Both the ${\mathrm{MSC}}_{T}$ method and the modular reasoning are based on reasoner HermiT, and they are not parallelized but instead running in an arbitrary *sequential* order of ${\mathrm{MSC}}_{T}$'s or ABox partitions.

##### Queries

**Figure 4:**
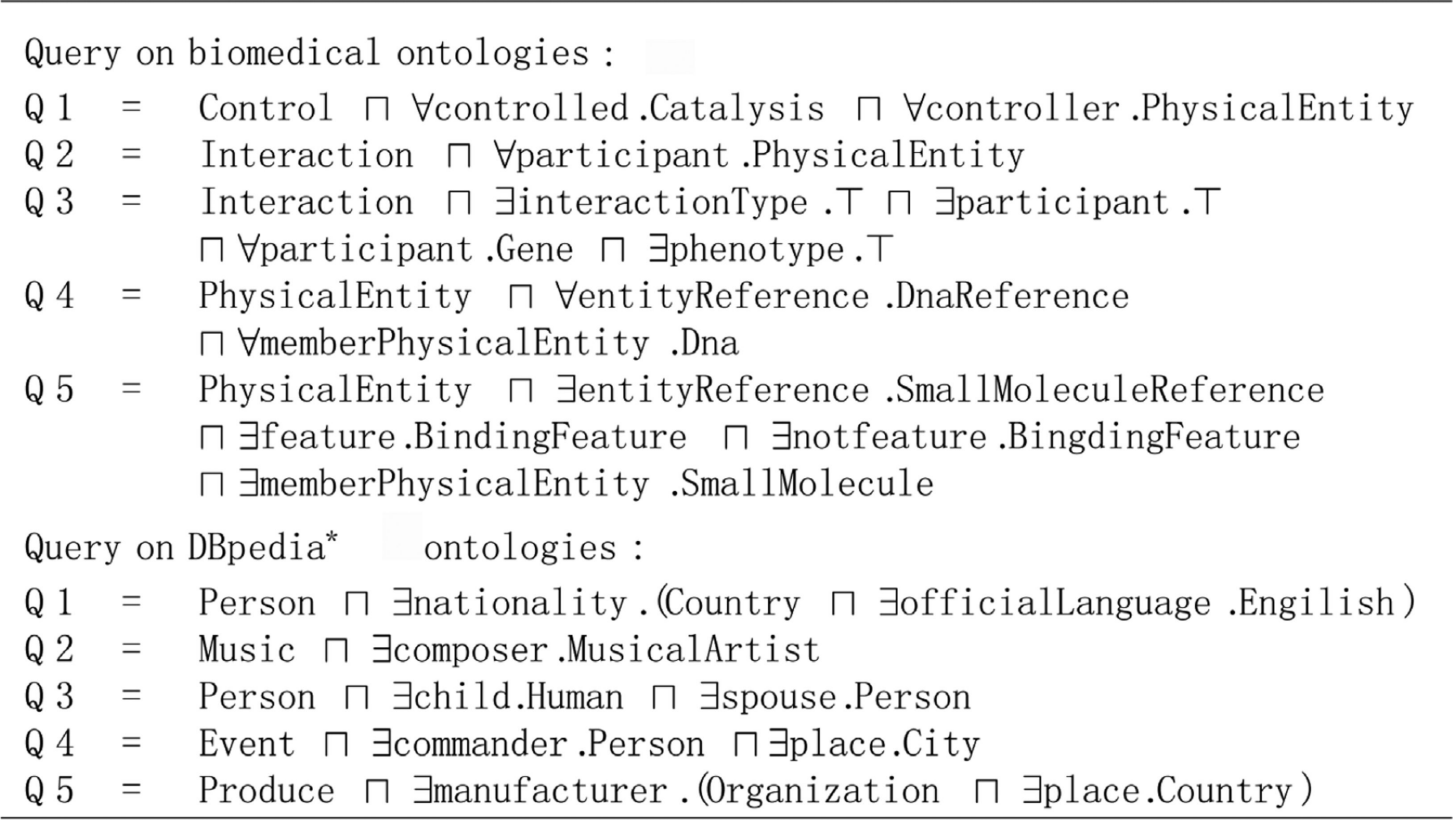
Queries for biomedical and DBpedia* ontologies.

LUBM comes with 14 standard queries. For biomedical and DBpedia* ontologies respectively, queries listed in **Figure 4** are used.

**Figure 5:**
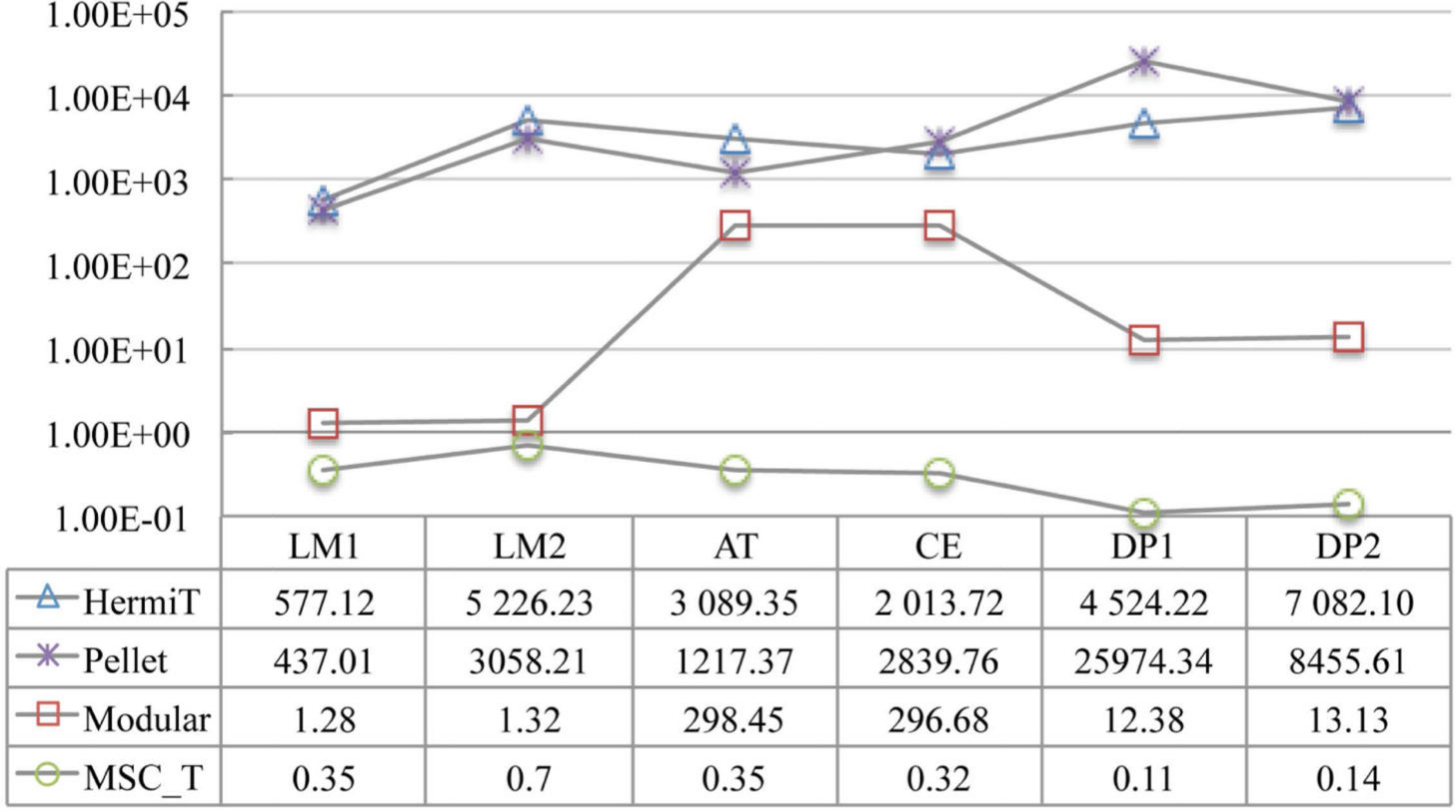
Average time (ms) on instance checking.

**Figure 6:**
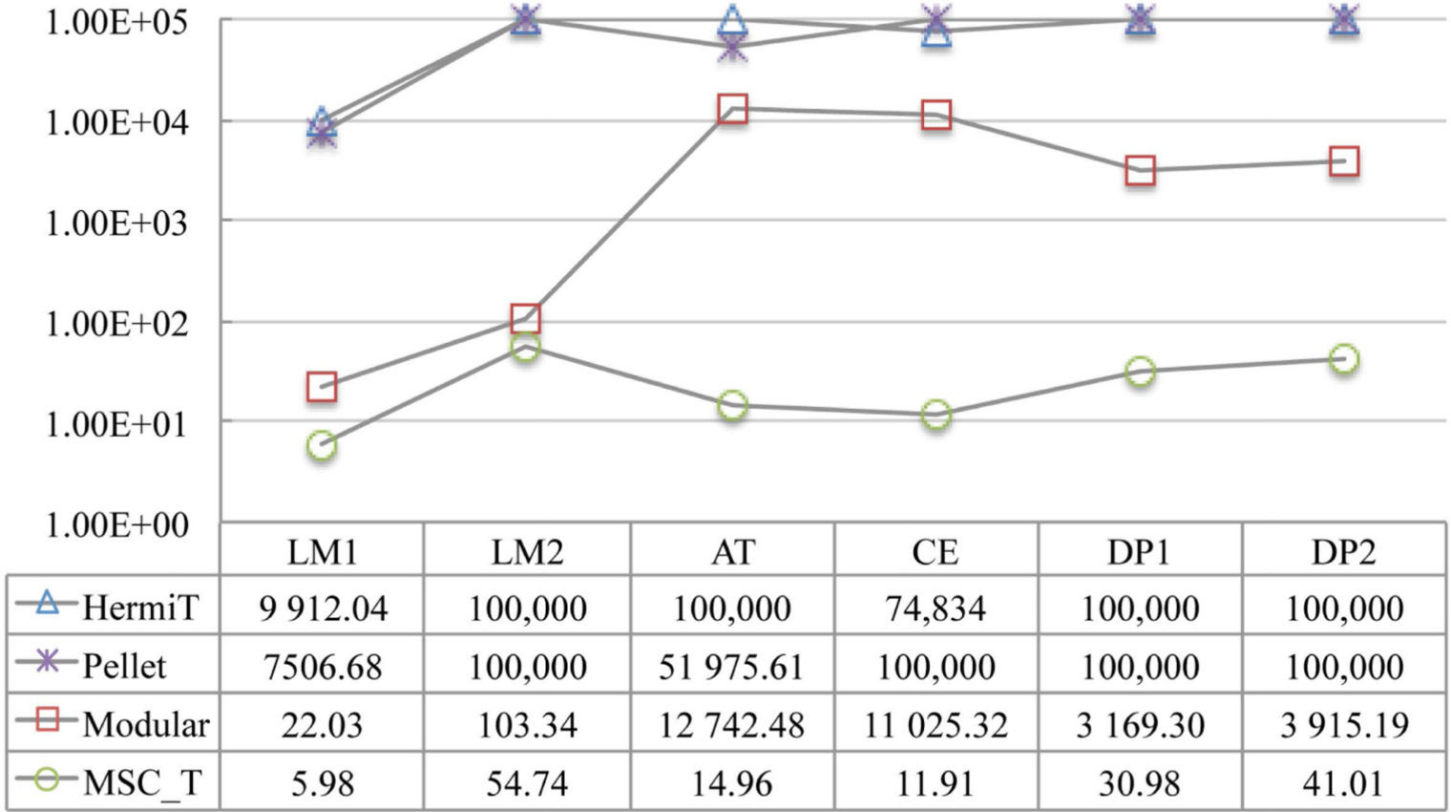
Average time(s) spent on instance retrieval. Timeout is set to be 100,000 s.

For each test ontology, we run the reasoning for each of the given queries. We report the average reasoning time spent on instance checking (**Figure 5**) and instance retrieval (**Figure 6**), respectively. The reasoning time reported here does not include the time spent for resource initialization (*i.e.* ontology loading and reasoner initialization), since the initialization stage can be done offline for query answering. However, it is obvious that the ${\mathrm{MSC}}_{T}$ method should be more efficient, since it only requires to load an ontology TBox while a regular ABox reasoning requires to load an entire ontology (including large ABoxes). For reasoning with ${\mathrm{MSC}}_{T}$'s and ABox partitions, any updates during the query answering procedure (e.g. update the reasoner for different ABox partitions or different ${\mathrm{MSC}}_{T}$'s) is counted into the reasoning time.

Another point worth noting here is that, for answering object queries using either modular reasoning or the ${\mathrm{MSC}}_{T}$ method, the overhead (time for ABox modularization or computation of ${\mathrm{MSC}}_{T}$'s) should be taken into account. However, as shown in previous section and in [16], this overhead is negligible comparing with the efficiency gained on the reasoning, not to mention when these two methods get parallelized using existing frameworks such as MapReduce [45].

#### 6.2.2. Result Analysis

As can be seen from the above two figures, using the ${\mathrm{MSC}}_{T}$ method, reasoning efficiency for both instance checking and instance retrieval in the testing ontologies has been improved significantly: 1) by more than three orders of magnitude when comparing with a complete reasoning; 2) and by about two orders of magnitude (except in LUBM1 and LUBM2) when comparing with the modular reasoning. For the latter, the improvement in LUBM1 and LUBM2 are not as significant as in others, which is because of the simplicity of these two ontologies that allows fine granularity of ABox partitions to be achieved [45].

On the other hand, using the ${\mathrm{MSC}}_{T}$ method in complex ontologies, such as AT and CE, the great improvement in reasoning efficiency comes from the reduction of searching space for reasoning algorithms, by branch pruning and also *concept absorption* during the computation of ${\mathrm{MSC}}_{T}$'s. For example, consider an individual *x* having the following *n* role assertions:

$$R\left(x,y_{1}\right),R\left(x,y_{2}\right),\cdots ,R\left(x,y_{n}\right)$$

where *y_i_* ∈ *D* and *n* tends to be large in these practical ontologies. Rolling up these assertions may generate a set of ∃*R.D* 's, the conjunction of which is still ∃*R.D* . Thus, when using this concept for instance checking, the interpretation may generate only one *R* -neighbor of individual *x* instead of *n* .

### 6.3. Scalability Evaluation

**Figure 7:**
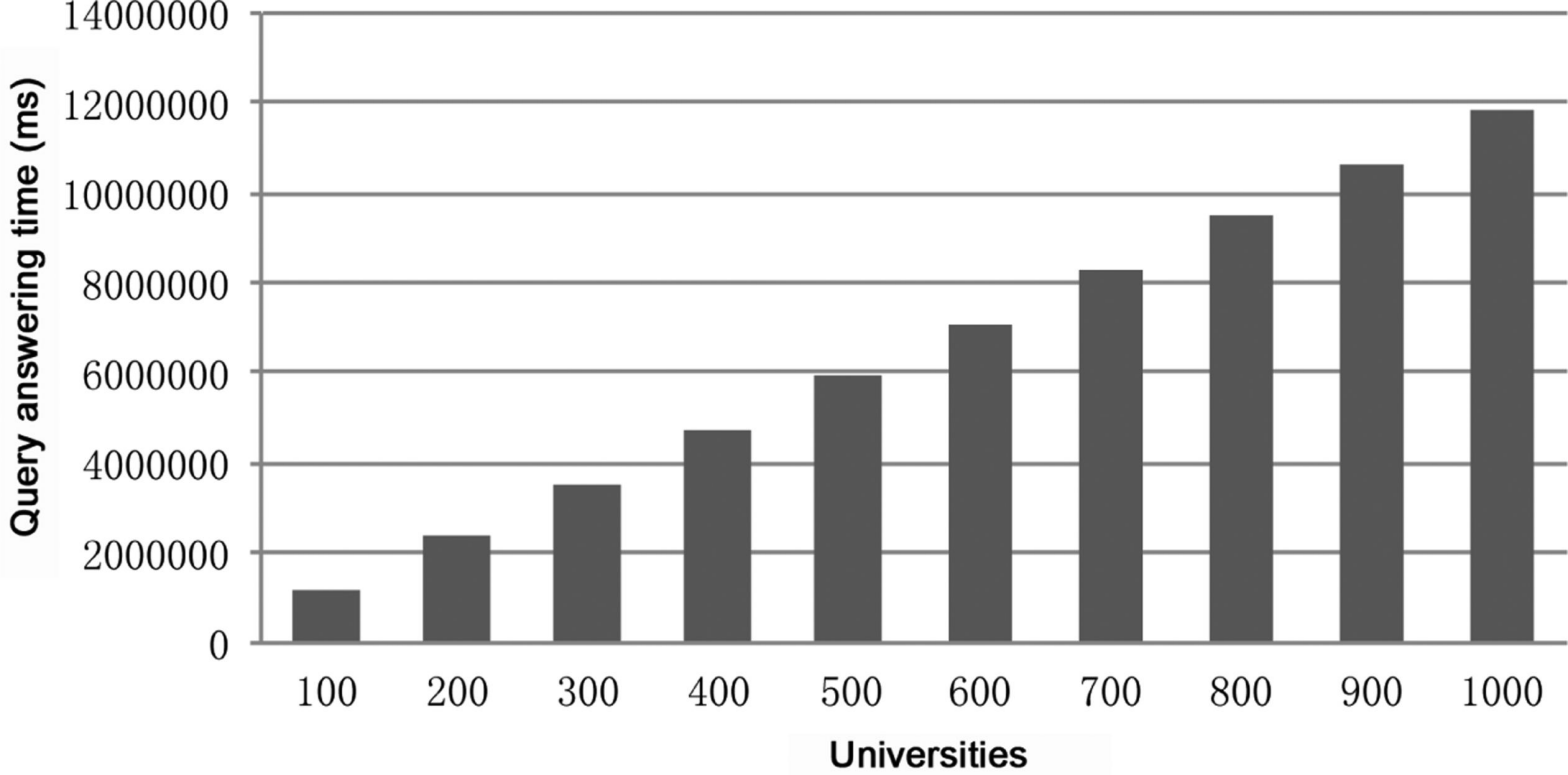
Scalability evaluation.

Using the ${\mathrm{MSC}}_{T}$ method for query answering over large ontologies is intended for distributed (parallel) computing. However, even if it is executed sequentially in a single machine, linear scalability may still be achieved on large ontologies that are not extremely complex; and there are mainly two reasons for that: first, the computation of ${\mathrm{MSC}}_{T}$'s focuses on only the query-relevant assertions instead of the entire ABox; second, the obtained ${\mathrm{MSC}}_{T}$'s could be very simple, sizes of which could be significantly smaller than that of the ABoxes. We test the scalability of this method for query answering (sequentially executed) using the benchmark ontology LUBM, which models organization of universities with each university constituted about 17,000 related individuals. The result is show in **Figure 7**.

## 7. Conclusions and Outlook

In this paper, we proposed a revised MSC method for efficient instance checking. This method allows the ontology reasoning to explore only a much smaller subset of ABox data that is relevant to a given instance checking problem, thus being able to achieve great efficiency and to solve the limitation of current memory-based reasoning techniques. It can be particularly useful for answering object queries over those large *non-Horn* DL ontologies, where existing optimization techniques may fall short and answering object queries may demand thousands or even millions of instance checking tasks. Most importantly, due to the independence between ${\mathrm{MSC}}_{T}$'s, scalability for query answering over huge ontologies (e.g. in the setting of semantic webs) could also be achieved by parallelizing the computations.

Our technique currently works for logic SHI , which is semi-expressive and is sufficient for many of the practical ontologies. However, the use of more expressive logic in modeling application domains requires more advanced technique for efficient data retrieving from ontology ABoxes. For the future work, we will investigate on how to extend the current technique to support SHIN or SHIQ that are featured with (qualified) number restrictions. We will concentrate on extending the rolling-up procedure to generate number restrictions, such as ≥ *nR*.T or ≥ *nR.C* , whenever there is a need. We will also have to take a particular care of the identical individual problem, where concepts and role assertions of an individual can be derived via individual equivalence.

## Figures and Tables

**Table 1:** Information of tested ontologies.

Ontology	Expressivity	# Cpts.	# Axms.	# Ind.	# Ast.
LM1	SHI	43	42	17,175	67,465
LM2	SHI	43	42	78,579	319,714
AT	SHI	59	344	42,695	117,405
CE	SHI	59	344	37,162	105,238
DP1	SHI	449	465	273,663	402,062
DP2	SHI	449	465	298,103	419,505

**Table 2:** Quantification depth of ${\mathrm{MSC}}_{T}$'s from different rolling-up procedures.

	V1	V2	V3

Ontology	Max./Avg.	Max./Avg.	Max./Avg.
LM1	5103/4964.68	215/98.8	2/1.48
LM2	23,015/22654.01	239/103.59	2/1.51
AT	2605/2505.50	1008/407.97	8/3.02
CE	3653/3553.4	1106/437.18	8/2.76
DP1	3906/3070.80	50/2.98	4/1.13
DP2	3968/3865.60	58/2.94	5/1.12

**Table 3:** Number of conjuncts of ${\mathrm{MSC}}_{T}$'s from different rolling-up procedures.

	V1	V2	V3
LM1	104/31.34	8/3.58	4/1.56
LM2	203/64.71	8/3.78	4/1.59
AT	88/87.99	19/5.92	13/1.97
CE	52/50.70	16/6.25	17/1.94
DP1	33,591/26864.50	71/5.19	12/1.83
DP2	60,047/60011.60	64/3.95	12/1.79

## Footnotes

1 Note that, to follow the simple-form concept restriction, multiple axioms may be added.

3 Note that ∃*R.C* ⊑ *D* is equivalent with $\exists R^{-}.\neg D⊑\neg C$ .

4 http://sourceforge.net/projects/owlapi

5 http://www.reactome.org/download
